# Anaphylaxis due to midazolam administered before induction of general anesthesia: a case report

**DOI:** 10.1186/s40981-025-00800-0

**Published:** 2025-06-19

**Authors:** Ryosuke Funabiki, Tatsuo Horiuchi, Toshie Shiraishi, Masaki Orihara, Kazuhiro Nagumo, Shigeru Saito

**Affiliations:** 1 Department of Anesthesiology, Gunma University Graduate School of Medicine, 3-39-22 Showa-machi, Maebashi, Gunma 371-8511 Japan; 2 Department of Anesthesiology, Gunma University Hospital, 3-39-15, Showa-machi, Maebashi, Gunma 371-8511 Japan; 3 Department of Anesthesiology, Yotsuya Medical Cube, 7-7 Niban-cho, Chiyoda-ku, Tokyo, 102-0084 Japan; 4 Intensive Care Unit, Gunma University Hospital, 3-39-15, Showa-machi, Maebashi, Gunma 371-8511, Japan

**Keywords:** Anaphylaxis, Midazolam, Skin tests

## Abstract

**Background:**

Anaphylaxis is an immediate allergic reaction. However, in some cases, there is a delay between the administration of the causative agent and the onset of anaphylaxis.

**Case presentation:**

A 41-year-old woman was scheduled for laparoscopic myomectomy under general anesthesia combined with epidural anesthesia. Midazolam was administered, and an epidural catheter was inserted. Seven minutes after the induction of general anesthesia (17 min after midazolam administration), the patient developed tachycardia, hypotension, and redness of the face and trunk. Her hemodynamic status improved after administration of phenylephrine and elevation of both legs, and the surgery was completed. Increased blood histamine and tryptase levels were observed 30 min after the onset of hemodynamic signs. Based on the above, anaphylaxis was diagnosed. Skin tests later showed that midazolam was the causative agent.

**Conclusions:**

A case of perioperative anaphylaxis caused by midazolam, which was used before the induction of general anesthesia, was described.

## Background

Many anaphylaxis cases occur within several minutes after administering the causative agent. However, there have been several cases in which anaphylaxis do not occur immediately after administration of the causative agent. A case of anaphylaxis due to midazolam administered during epidural catheter insertion, which developed after the induction of general anesthesia, is reported.

## Case presentation

A 41-year-old woman (height 159.6 cm, weight 62.5 kg) was scheduled for laparoscopic myomectomy for uterine fibroids under general anesthesia combined with epidural anesthesia. Preoperative conditions included hay fever. She had had a previous cesarean section and no complications with spinal anesthesia. Her history of benzodiazepine use was unknown.

**Fig. 1:**
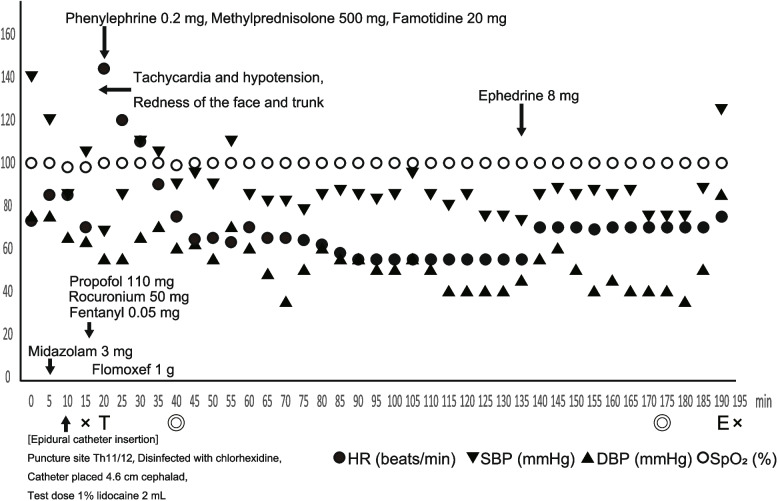
Changes in vital signs and drug administration during anesthesia. Seven minutes after induction of general anesthesia (17 min after intravenous administration of midazolam), the patient develops tachycardia and hypotension. × : start of anesthesia (left side), end of anesthesia (right side), T: tracheal intubation, E: tracheal extubation, ◎: start of surgery (left side), end of surgery (right side), SBP: systolic blood pressure, DBP: diastolic blood pressure, HR: heart rate, SpO_2_: percutaneous oxygen saturation. The *X*-axis represents time, and the *Y*-axis represents HR (beats·min.^−1^), BP (mmHg), and SpO_2_ (%)

When the patient came to the operating room, midazolam 3 mg was administered intravenously. After placing the patient in a side-lying position, her back was disinfected with chlorhexidine. Then, an epidural catheter was inserted 4.6 cm cephalad from the level of Th 11/12, and 2 mL of 1% lidocaine was administered epidurally as a test dose under mild sedation. There were no findings to suggest intravascular or subarachnoid placement of the epidural catheter. General anesthesia was induced with 110 mg propofol, 0.05 mg fentanyl, and 50 mg rocuronium under oxygen administration. Her blood pressure and heart rate before the onset of hypotension and tachycardia were 105/63 mmHg and 70 beats·min^−1^, respectively. Seven minutes after induction of general anesthesia (17 min after midazolam administration), her blood pressure decreased to 68/55 mmHg, and her heart rate increased to 144 beats·min^−1^. Redness appeared on the face and trunk, but there were no wheals. There was no airway pressure increase. Her hemodynamic status improved gradually after administration of phenylephrine 0.2 mg and elevation of both legs. Subsequently, 500 mg of methylprednisolone and 20 mg of famotidine were administered. Adrenaline was prepared, but it was not administered because her condition improved. After the anesthesiologists and surgeons determined that her condition was stable, that the causative agent might be rocuronium because rocuronium was the most common causative agent of perioperative anaphylaxis, and that epidural anesthesia could replace the rocuronium, the surgery started. Twenty minutes after the start of surgery, continuous epidural anesthesia (100 mL 0.2% ropivacaine, 0.4 mg fentanyl, 5 mg droperidol, total 110 mL; administration rate 4 mL·h^−1^) was started. No additional rocuronium was administered because the possibility that rocuronium was the cause of the anaphylaxis was considered. Intraoperative respiratory and circulatory parameters were stable (Fig. 1). After the surgery, she was extubated in the operating room and left the room. The operative time was 2 h and 13 min, and the anesthesia time was 2 h and 59 min. The clinical score for suspected perioperative immediate hypersensitivity reactions was 14 points [1] (Table 1). This score is designed to assess the likelihood of perioperative anaphylaxis and is based on the sum of the scores of the following five items: cardiovascular symptoms, respiratory symptoms, skin symptoms, combination of symptoms, and time from administering the suspected drug to the appearance of symptoms [1] (Table 1). Each item is assigned a score based on severity or related criteria, and the total score is interpreted across five levels. In the present case, the total score was 14, which corresponds to the third level out of five.

**Table 1:** Clinical score

	Score
1. Cardiovascular	+ 6
2. Respiratory	0
3. Dermal/mucosal	+ 3
4. Combination	+ 5
5. Timing	0
Total	**14**

Fourteen points in total, likely to be an immediate hypersensitivity reaction [1]

A blood test 30 min after the onset of anaphylaxis showed histamine at 1.75 ng·mL^−1^ (diagnostic threshold 1.5 ng·mL^−1^ [2]) and tryptase at 9.1 µg·L^−1^. Blood tests performed 24 h after onset showed histamine at 0.88 ng·mL^−1^ and tryptase at 4.8 µg·L^−1^. The threshold of tryptase calculated on this baseline was 7.76 µg·L^−1^ [3, 4]. After the onset of anaphylaxis, blood histamine and tryptase concentrations were both above the diagnostic thresholds [2, 3], supporting the diagnosis of anaphylaxis.

**Fig. 2:**
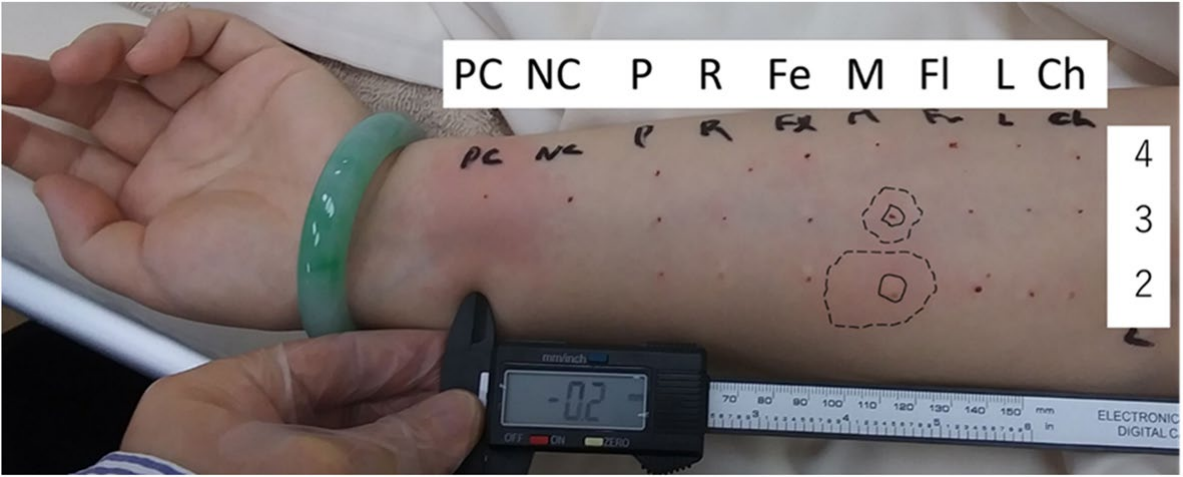
Results of intradermal tests performed 39 days after surgery. Solid lines and dashed lines indicate wheals and erythema, respectively. Only midazolam shows a positive reaction. PC: positive control, NC: negative control, P: propofol, R: rocuronium, Fe: fentanyl, M: midazolam, Fl: flomoxef, L: lidocaine, Ch: chlorhexidine. The 4, 3, and 2 on the right side of the figure represent the concentrations of the drug. PC-4: 10 μg·mL^−1^, NC-4: 9000 μg·mL^−1^, P-4: 10 μg·mL^−1^, P-3: 100 μg·mL^−1^, P-2: 1000 μg·mL^−1^, R-4: 0.5 μg·mL^−1^, R-3: 5 μg·mL^−1^, R-2: 50 μg·mL^−1^, Fe-4: 0.05 μg·mL^−1^, Fe-3: 0.5 μg·mL^−1^, Fe-2: 5 μg·mL^−1^, M-4: 5 μg·mL^−1^, M-3: 50 μg·mL^−1^, M-2: 500 μg·mL^−1^, Fl-4: 20 μg·mL^−1^, Fl-3: 200 μg·mL^−1^, Fl-2: 2000 μg·mL^−1^, L-4: 10 μg·mL^−1^, L-3: 100 μg·mL^−1^, L-2: 1000 μg·mL^−1^, Ch-4: 0.02 μg·mL^−1^, Ch-3: 0.2 μg·mL^−1^, Ch-2: 2 μg·mL.^−1^

Thirty-nine days after the onset of anaphylaxis, skin tests were performed to identify the causative agent. The test drugs were histamine (10 mg·mL^−1^ for the prick test and 10 µg·mL^−1^ for the intradermal test) as the positive control, saline solution as the negative control, and propofol, rocuronium, fentanyl, midazolam, flomoxef sodium, lidocaine, and chlorhexidine as suspect drugs. The maximum concentrations of agents used for skin testing and the diagnostic criteria were determined according to several perioperative anaphylaxis guidelines [3, 5, 6]. According to Société Française d’Anesthésie et de Réanimation (SFAR)-Société Française d’Allergologie (SFA) and Japanese Society of Anesthesia (JSA) criteria, a positive prick test result is defined as the appearance, after 20 min, of a wheal that has a diameter that is 3 mm greater than that of the negative control or a diameter of at least half the diameter of the positive control wheal. For an intradermal test, between 0.02 and 0.05 mL of the suspect drug are injected into the skin [6]. The criterion for a positive intradermal test result is the appearance after 20 min of an erythematous wheal, the diameter of which is at least equal to twice that of the postinjection wheal [5, 6]. The prick test was positive only for histamine, and the intradermal test was positive for histamine and midazolam 50 µg·mL^−1^ and 500 µg·mL^−1^ (Fig. 2, Table 2). Based on these results, the composite score according to the Japan Epidemiological Study of Perioperative Anaphylaxis (JESPA) was 3 points, which indicated perioperative anaphylaxis. Therefore, the patient was diagnosed with anaphylaxis caused by midazolam.

**Table 2:** Results of intradermal tests

Drug	Concentration (μg・mL^−1^)	Erythema (mm)	Wheals (mm)	Diagnosis
Positive control	10	32	16	**Positive**
Negative control	9000	0	3	**Negative**
Propofol	10	0	3	**Negative**
Propofol	100	0	3	**Negative**
Propofol	1000	0	3	**Negative**
Rocuronium	0.5	0	3	**Negative**
Rocuronium	5	0	3	**Negative**
Rocuronium	50	0	3	**Negative**
Fentanyl	0.05	0	3	**Negative**
Fentanyl	0.5	0	3	**Negative**
Fentanyl	5	0	3	**Negative**
Midazolam	5	0	3	**Negative**
Midazolam	50	15	6	**Positive**
Midazolam	500	23	7	**Positive**
Flomoxef	20	0	3	**Negative**
Flomoxef	200	0	3	**Negative**
Flomoxef	2000	0	3	**Negative**
Lidocaine	10	0	3	**Negative**
Lidocaine	100	0	3	**Negative**
Lidocaine	1000	0	3	**Negative**
Chlorhexidine	0.02	0	3	**Negative**
Chlorhexidine	0.2	0	3	**Negative**
Chlorhexidine	2	0	3	**Negative**

Saline and histamine were used as the negative and positive controls, respectively. Saline was used to dilute the drugs. The injection volume for the intradermal test was between 0.02 and 0.05 mL for all drugs, and a 3-mm wheal was made on the patient’s forearm. The diameters of the wheal and erythema were measured 20 min after injection, and a reaction was considered positive if the wheal diameter was equal to or greater than 6 mm. Midazolam 50 and 500 μg·mL^**−**1^ showed positive reactions

## Discussion

In the present case, anaphylaxis occurred after the induction of general anesthesia, and the causative agent was midazolam administered during epidural anesthesia before the induction of general anesthesia.

Perioperative anaphylaxis is often different from non-perioperative anaphylaxis, and there are many differential diagnoses [6]. Diagnosing perioperative anaphylaxis requires a combination of several methods in addition to the diagnostic criteria given by the World Allergy Organization (WAO) [7]. In the present case, the clinical score [1] was used to diagnose perioperative anaphylaxis.

The clinical score is based on the sum of the scores of the following five items: cardiovascular symptoms, respiratory symptoms, skin symptoms, combination of symptoms, and time from administering the suspected drug to the appearance of symptoms. Because the clinical score for this case was 14 points in total, likely to be an immediate hypersensitivity reaction [1], perioperative anaphylaxis was suspected. However, perioperative anaphylaxis could not be diagnosed based on the clinical score alone. Therefore, histamine, tryptase, and skin test results were used to diagnose perioperative anaphylaxis.

There are currently several diagnostic thresholds of tryptase for perioperative anaphylaxis. Recently, the tryptase threshold specified by the European Academy of Allergy and Clinical Immunology (EAACI), calculated as 1.2 × baseline tryptase + 2 µg·L^−1^ [3], has shown high accuracy [8, 9]. Furthermore, the histamine threshold for perioperative anaphylaxis also has high accuracy [2]. In the present case, both tryptase and histamine levels were above the thresholds.

Skin testing is essential to diagnose perioperative anaphylaxis and identify the causative agent. According to the JSA guideline, all agents used before the onset of anaphylaxis should be tested [6]. Generally, rocuronium and antibiotics, usually used during general anesthesia induction, are the common causative agents of perioperative anaphylaxis [6, 10–12]. In addition, chlorhexidine, the antiseptic used most often in the perioperative period, has been reported as the third most common cause of perioperative anaphylaxis in a recent large-scale study conducted in the 6th National Audit Project (NAP6) [11]. However, midazolam, which was used before the induction of general anesthesia, was the causative agent of perioperative anaphylaxis in the present case. The present case showed that the causative agent of perioperative anaphylaxis should not be identified only by the timing of the onset of anaphylaxis.

However, other agents used in the skin tests may have shown false-negative results. Therefore, duplicate skin tests could have been done to confirm the diagnosis.

Skin testing should be administered within the maximum concentrations indicated in the guidelines to avoid false-positive results [6]. The maximum concentration of midazolam (intradermal test) was 500 μg·mL^−1^ in the 2011 guideline in France and the 2021 guideline by the JSA for perioperative anaphylaxis [5, 6], whereas a concentration of 50 μg·mL^−1^ is recommended in the 2019 guideline for perioperative anaphylaxis [3]. Although the intradermal test was positive for midazolam at both 50 and 500 μg·mL^−1^ in the present case, anesthesiologists should be aware of the concentrations of suspected agents for skin testing. In addition, skin testing carries a risk of triggering anaphylaxis [6]. In the present case, midazolam 50 μg·mL^−1^ was positive on the intradermal test. Therefore, we did not have to test midazolam 500 μg·mL^−1^ to avoid the recurrence of anaphylaxis that may occur during the test. Furthermore, the intradermal test result of 500 µg·mL^−1^ midazolam might cause a false-positive result.

In addition, the composite score specified by JESPA [13] was used for diagnosis. The composite score is determined by summing the scores of three items: (1) clinical score, (2) tryptase level, and (3) skin test and basophil activation test results. A score of 2 or more indicates perioperative anaphylaxis. The composite score for the present case was 3 points, indicating perioperative anaphylaxis. The composite score may be helpful when perioperative anaphylaxis is difficult to diagnose with a clinical score of 21 or less, as in the present case.

In the present case, the timing from midazolam administration to the onset of anaphylaxis was 17 min. The median time to the onset of symptoms of anaphylaxis caused by midazolam is 2 min [14]. Regarding the clinical score, shorter intervals between administration of the suspected agent and symptom onset are associated with higher scores. Specifically, 5 and 15 min are the criteria for the time from administering the causative agent intravenously to the onset of anaphylaxis [1]. This is particularly relevant for intravenous drugs, since the rapid onset of symptoms is considered to support the likelihood of causality. In the present case, midazolam was administered intravenously before inserting the epidural catheter, and the patient was sedated during the epidural catheter insertion. Therefore, midazolam was unlikely to remain in the intravenous drip route until the onset of anaphylaxis.

Several reports have shown that anaphylaxis does not develop immediately after midazolam administration. The time from administering midazolam intramuscularly to the onset of anaphylaxis was 30 min [15]. The time from administering midazolam intranasally to the onset of anaphylaxis was 11 min [16]. The onset of anaphylaxis caused by non-intravenous causative agents might be delayed.

However, there is a case in which the time from administering midazolam to the onset of anaphylaxis was 42 min [14]. Although the cause of this is unclear, several cases showed that anaphylaxis does not always develop immediately after administering the causative agent; further studies might show their epidemiological and other characteristics.

This report had several limitations. First, substances such as remimazolam were not tested in the skin test, which has been reported to exhibit cross-reactivity with midazolam [17]. Since the present patient might have examinations under sedation in the future, a search for alternative medications should be conducted. We would like to perform further tests if the patient wishes to test for cross-reactivity with other benzodiazepines.

Second, decimal values were not noted in the skin tests. Although we believe the skin test results were reliable, we will record the decimal values in future studies.

Third, adrenaline was not administered in this case. The first-line treatment for anaphylaxis is adrenaline [6], which should be administered early once symptoms of anaphylaxis have been recognized or suspected [18]. Thus, we should treat future anaphylaxis cases with adrenaline as soon as possible.

A case of perioperative anaphylaxis caused by midazolam, which was used before the induction of general anesthesia, was described. A definitive diagnosis of midazolam-induced anaphylaxis was supported by a non-low clinical score, positive findings on skin testing and serological evaluations, and a high score on the recently proposed composite scoring system.

## Data Availability

The datasets used during the current case are available from the corresponding author upon reasonable request.

## Abbreviations

SFAR: Société Française d’Anesthésie et de Réanimation
SFA: Société Française d’Allergologie
JSA: Japanese Society of Anesthesiology
JESPA: Japan Epidemiological Study of Perioperative Anaphylaxis
WAO: World Allergy Organization
EAACI: European Academy of Allergy and Clinical Immunology
NAP6: 6Th National Audit Project
